# Death from Chloroform

**Published:** 1866-04

**Authors:** 


Death from Chloroform.—Another death from chloroform has recently taken place at the St. Mary's Hospital, Dublin. From the evidence at the inquest, it appears that all proper precautions had been taken, and no cause for the sudden syncope could be detected on post mortem examination. The chloroform had been given preparatory to the operation of evulsion of toe-nail.

